# Comparative Study of Laterite and Bentonite Based Organoclays: Implications of Hydrophobic Compounds Remediation from Aqueous Solutions

**DOI:** 10.1155/2013/681769

**Published:** 2013-11-05

**Authors:** Muhammad Nafees, Amir Waseem, Abdur Rehman Khan

**Affiliations:** ^1^Department of Inorganic Chemistry, Nanjing University, Nanjing 210093, China; ^2^Department of Chemistry, COMSATS Institute of Information Technology, Abbottabad 22060, Pakistan; ^3^Department of Chemistry, Quaid-i-Azam University, Islamabad 45320, Pakistan

## Abstract

Four cost effective organoclays were synthesized, characterized, and studied for the sorption of hydrophobic compounds (edible oil/grease and hydrocarbon oil) from aqueous solutions. Organoclays were prepared by cation exchange reaction of lattice ions (present onto the surface of laterite and bentonite clay minerals) with two surfactants, hexadecyl trimethyl ammonium chloride (HDTMA-Cl) and tetradecyl trimethyl ammonium bromide (TDTMA-Br). Fourier transform infrared spectroscopy and scanning electron microscopy were used for the characterization of synthesized organoclays. It was found that the amount of surfactant loading and the nature of the surfactant molecules used in the syntheses of organoclay strongly affect the sorption capacity of the clay mineral. Further, it was found that both the laterite and bentonite based organoclays efficiently removed the edible and hydrocarbon oil content from lab prepared emulsions; however, the adsorption capacity of clay mineral was greatly influenced by the nature of hydrophobic compounds as well.

## 1. Introduction

Across Pakistan, surface and groundwater sources continue to be polluted by raw sewage, industrial waste, and agricultural runoff. Less than half of the urban sewage is drained off through sewers and covered drains, and only a small fraction of that is treated before being disposed of into water bodies [1–3]. The pressure on water resources due to industrial growth is quite significant and has increased water pollution problems. In Pakistan, a small fraction of wastewater is being treated (less than 1%) by industries due to high wastewater treatment costs and it is common practice for wastewater to be discharged directly into fresh water resources. For example, in KPK province, 80,000 m^3^ of industrial effluents containing a very high level of pollutants is discharged into the streams and rivers on a daily basis causing skin diseases and loss in agricultural productivity and fish population [4]. The edible oil industry and car wash stations discharge poor quality effluents, which are a serious threat to water resources in Pakistan.

Remediation of environmental contaminants is a great challenge for the modern world as an increasing global population is creating a burden on industries to supply output that meets demands [2]. In the developing world this demand is often met at the expense of the environment as regulations are not usually adhered to by industries. Layered silicate clay minerals are naturally occurring earth, which can be value added by modifying their adsorption properties to enhance their efficiency in removing contaminants that can have a detrimental environmental impact [5]. Naturally occurring clay minerals such as smectite can be modified by the introduction of cationic surfactants such as quaternary ammonium compounds into the clay structure to make them hydrophobic. These clays are then referred to as organoclays and are suitable for a large number of environmental remediation applications [6]. It has been found that the structure and chemical characteristics of the clay mineral have a strong effect on the cation exchange that is involved in the syntheses of organoclays [7]. Usually the cation exchange reaction starts at the edges of the clay particle and then extends to the center. Kinetic studies show that the reaction between the quaternary ammonium salts and the clay mineral increases with the increase of temperature [8]. Organoclays are usually divided into two groups depending on the organic cation and sorption mechanism. The first group is the adsorptive organoclays which consist of short chain quaternary ammonium ion (R ≤ 12), such as TMA (tetramethyl ammonium) and TBA (tetrabenzyl ammonium). The second type of organoclays is organophilic clays which consist of a long chain quaternary ammonium ion (R ≥ 12) such as HDTMA (hexadecyl trimethyl ammonium) and ODTMA (octadecyl trimethyl ammonium) [9]. Organoclays have been used as sorbents in many industrial and environmental applications [10–12]. Studies have shown that modification of the clay mineral with organic cation greatly enhances the properties of the clay mineral to be used for the removal of organic/inorganic contaminants from water and wastewater. HDTMA bentonite based organoclays were used for the removal of textile dyes [13–15], benzene [16], tannins [17], phenols [18], nitrate [19], dibenzofuran [20], hexavalent chromium [21], acid orange 10 and Pb(II) ions [22], nitrobenzoic acid [23], arsenic [24], BTEX, and phenol [25].

The synthesis of organoclays from cheap raw material is simple and very cost effective as organoclays are less expensive than other existing materials or nanomaterials, because the basic materials come from readily available natural sources and because they are produced in existing, full-scale production facilities (for commercially available organoclays). In this paper, we investigated the sorption of edible oil and hydrocarbon (HC) oil water emulsion by Pakistani laterite and bentonite clay, modified with HDTMA-Cl and TDTMA-Br. This is the first study using laterite and bentonite based organoclays of Pakistani origin for the removal of edible and HC oil contents from aqueous solutions. Sorption of edible and HC oil content onto the synthesized organoclays was performed on lab prepared emulsions using different sorbent concentrations in order to evaluate the maximum sorption capacity of edible and HC oil. Possible uses of these organoclays are for the removal of oil/grease and HC content from the discharges of the edible oil industry and car wash stations. 

## 2. Material and Methods

### 2.1. Characterization of the Clay Mineral

The clays used in this study were laterite from Haripur and bentonite from Swabi Khyber Pakhtunkhwa (KPK), Pakistan. The clay mineral was first characterized with X-ray fluorescence spectroscopy (PANalytical Cubix XRF, Model PW2300). Cation exchange capacity (CEC) of the clay mineral was measured using BaCl_2_ method [26]. 

### 2.2. Synthesis of Organoclays

Four long chain organoclays were prepared using bentonite and laterite clay minerals, while tetradecyl trimethyl ammonium bromide (C_17_H_38_N-Br, Merck, purity ≥ 98%) and hexadecyl trimethyl ammonium chloride (C_19_H_42_N-Cl, Merck, purity ≥ 98%) were used as organic modifier. The organoclays were synthesized using the previously employed procedure [27]. Briefly, 12.5 g of laterite and bentonite were separately taken in a volumetric flask and were mixed with a solution of quaternary ammonium salt equivalent to the cation exchange capacity (CEC) of the clay (84 cmol/kg of bentonite and 52 cmol/kg of laterite). The solution was than stirred for 24 hours at room temperature on a magnetic stirrer. The HDTMA and TDTMA modified laterite and bentonite clays were then washed with distilled water repeatedly until it became free of bromide and chloride ions as detected by AgNO_3_. The synthesized clays were then quickly frozen, freeze dried (labconco freezone 4.5 freeze drying instrument), and stored in a desiccator for later use. The organic carbon content (%OC) of the organoclays was measured using a carbon sulfur analyzer (leco CS-300) and was found to be 4.40% and 3.05% for HDTMA and TDMTA laterite, respectively. It was 5.5% and 4.90% for HDTMA and TDTMA bentonite, respectively. 

### 2.3. Organoclays Characterization

Organoclays were characterized by fourier transform infrared (FT-IR) spectroscopy using a Bruker FT-IR (Tensor 27) spectrometer in the range of 4000–500 cm^−1^ using KBr pallet and scanning electron microscopy (SEM Hitachi S-4800). 

### 2.4. Sorption of Edible Oil/Grease and Hydrocarbon Oil

Sorption of edible and hydrocarbon oil (HC, diesel, and engine oil) content on HDTMA and TDTMA laterite and bentonite based organoclays was performed using ASTM F716-09 [28]. Briefly, 5.0 g of any oil (edible or HC oil) was added to the deionized water (1000 mL) and the mixture was stirred on a magnetic stirrer for 1 hour at room temperature. HDTMA and TDTMA laterite and bentonite based organoclays were then added into lab prepared emulsions with a dose of about 0.2 to 1.0 g/L (twenty-five to five times lower than the oil content) and stirred again at a high speed for 30 min. After soaking for 30 min, both the organoclays were vertically transferred into a preweighed container and allowed to drain for 20 min. Subsequently, the saturated organoclays were transferred into another preweighed container and weighed. All samples were replicated three times and the mean values were taken for calculation. The percentage efficiency for the removal of oil and grease content was determined by subtracting the weight of organoclays with adsorbed oil (*C*_*s*_) from the original weight of organoclays (*C*_0_), dividing it by the original weight of organoclays (*C*_0_), and then multiplying it by 100.

(1) $$\begin{array}{c} \%\;\text{Removal}\;\text{Efficiency}=\frac{C_{s}-C_{0}}{C_{0}}\times 100. \end{array}$$

## 3. Results and Discussion

### 3.1. Characterization of Clay Mineral

The clay minerals were characterized using X-ray fluorescence spectroscopy. The results obtained are shown in Table 1. X-ray analysis shows that the laterite clay mineral was almost free of alkali earth metals and contains a high concentration of alumina and iron, while bentonite clay minerals contain both the alkaline and alkaline earth metals. Furthermore, the cation exchange capacity of clay minerals was measured using BaCl_2_ Method.

### 3.2. Organoclays Characterization Using Fourier Transform Infrared Spectroscopy (FT-IR)

**Figure 1:**
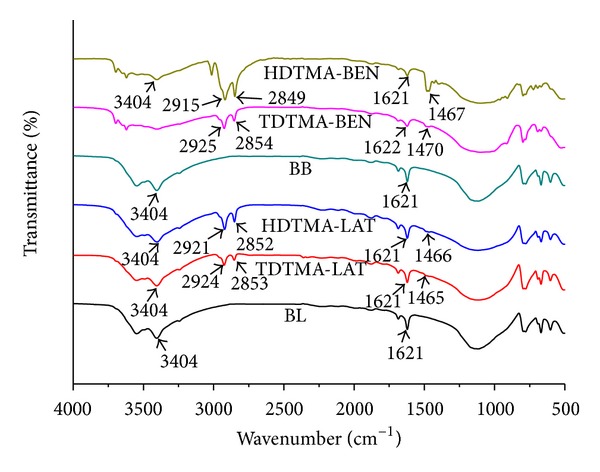
The FT-IR spectra of unmodified and modified laterite and bentonite with TDTMA and HDTMA (BL: blank laterite; BB: blank bentonite).

The FT-IR spectra of unmodified and modified laterite and bentonite with TDTMA and HDTMA were carried out over the range 500 to 4000 cm^−1^ and were compared with each other to obtain information on the modifications (Figure 1). The position and shape of the –OH stretching band in the IR spectra of bentonite minerals are basically influenced by the nature of the octahedral atoms to which the hydroxyl groups are coordinated [29]. A group of absorption peaks was observed between 3620 and 3404 cm^−1^, which is due to –OH stretching vibration bands of water in natural bentonite and HDTMA-bentonite and their bending vibrations at 910 and 918 cm^−1^, which is consistent with other studies [15, 30]. The absorption band at 3620 cm^−1^, found in the spectrum of natural bentonite, is typical for smectite minerals with large amounts of Al in the octahedral sheet [17, 20, 23, 29]. Another band at around 3404 cm^−1^ (stretching band of the –OH groups) was observed; the intensity of this band decreased with surfactant modification (see Figure 1) and is evidence for the modification of bentonite with surfactant (HDTMA) functional groups [24, 29]. The major differences arise when a pair of strong bands at 2849–53 and 2918–25 cm^−1^ was observed only in organoclays and can be assigned to the symmetric and asymmetric stretching vibrations of the methylene groups (*n*CH_2_) and their bending vibrations at 1465–70 cm^−1^, but these stretching bands are not observed in natural bentonite. This supports the modification of laterite and bentonite with surfactant cations [31]. The observed bands at 1621 cm^−1^ in both of the laterite and bentonite adsorbents, with and without modifications, also correspond to the –OH deformation of water.

### 3.3. Organoclays Characterization Using Scanning Electron Microscopy (SEM)

**Figure 2:**
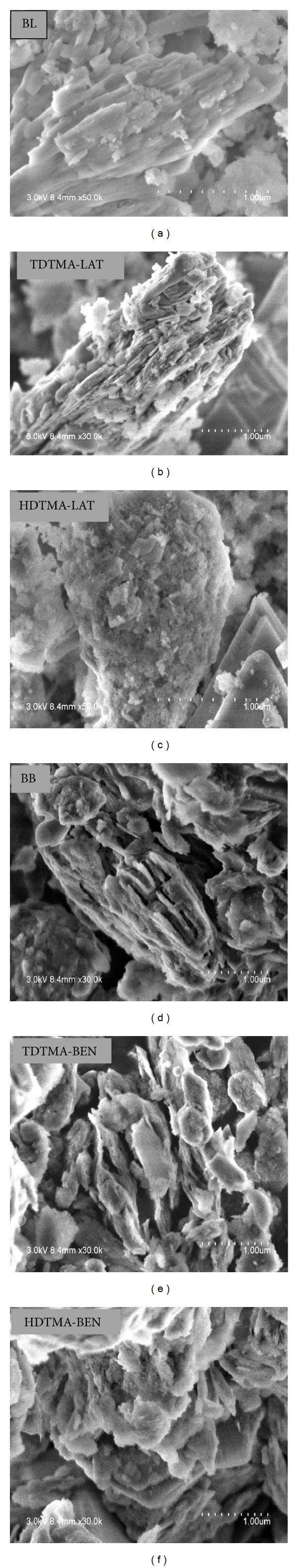
SEM micrographs of blank laterite (BL), TDTMA-LAT, HDTMA-LAT, blank bentonite (BB), TDTMA-BEN, and HDTMA-BEN (left to right).

It is stated that the organoclay strongly depends on the packing density of surfactant within the smectite interlayer spacing. At low surfactant concentrations, the intercalation of surfactant is usually random and the increase of surfactant packing density results in the regular stacking of surfactant-smectite galleries [32, 33]. It was found that not only the arrangement model of the surfactant, but also the morphologies of the organoclays strongly depended on the surfactant packing density within the interlayer [32–34]. The SEM images of laterite and bentonite before and after modification show that the surfactant reduces the particle size and aggregation, as we have a 1 : 1 CEC to surfactant loading, which is consistent with the previous studies [35]. It can be seen that the original clay has massive and curved plates (Figures 2(a) and 2(d)). Compared with the morphology of the laterite and bentonite, there are many small and aggregated particles and the plates become relatively flat in modified form (Figures 2(b), 2(c), 2(e), and 2(f)) forming nanophases with the potential for removing hydrophobic materials from water and wastewater.

### 3.4. Edible Oil and Hydrocarbon (Diesel and Engine Oil) Removal Efficiency of Organoclays

**Figure 3:**
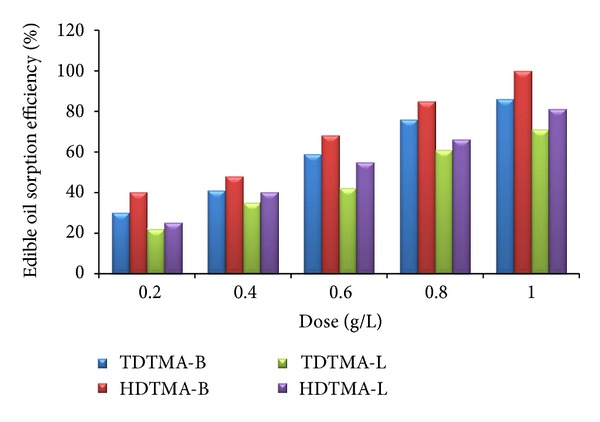
Removal efficiency of edible oil using HDTMA and TDTMA laterite and bentonite based Organoclays.

Both the HDTMA and TDTMA synthesized laterite and bentonite organoclays were used for the removal of edible oil and HC (hydrocarbon) oil content from lab prepared emulsions. The result, as shown in Figure 3 for edible oil sorption, indicates that the bentonite based HDTMA organoclays have removal efficiency of 100% for oil content and laterite based HDTMA organoclays have removal efficiency of 92% (at 1 : 5 for adsorbent to oil ratio). This is due to the high cation exchange capacity of the bentonite material which allows them to generate maximum Bronsted and Lewis acid activation sites. These activation sites provide a mean of attachment of organic alkyl ammonium salts such as HDTMA to the clay mineral. TDTMA synthesized laterite and bentonite organoclays have 86% removal efficiency of edible oil and grease content while laterite based organoclays have 71% (at 1 : 5 for adsorbent to oil ratio). In addition to edible oil sorption, both the HDTMA and TDTMA synthesized laterite and bentonite organoclays were used for the removal of hydrocarbon (diesel and engine oil) content from lab prepared emulsions. The study revealed that both the synthesized organoclays efficiently work for the hydrocarbon removal.  Table 2 gives the hydrocarbon sorption capacities of HDTMA and TDTMA synthesized laterite and bentonite organoclays. The hydrocarbon sorption capacity obtained for bentonite modified organoclay with either HDTMA or TDTMA is better than that obtained for the laterite based organoclays and is ranging from 2.4 to 5.2 g/g for diesel oil and 1.1 to 2.8 g/g for engine oil. The obtained results are slightly lower than those of the previously reported data [27], which may attribute to the difference in the nature of quaternary ammonium compound which ultimately results in lower adsorption capacity.

## 4. Conclusion

This study shows that the introduction of a new cost effective sorbent material (i.e., laterite and bentonite) and its modification toward long chain organoclays, which are synthesized by introducing organic molecules into the clay structure, is effective for the remediation of edible and HC oil contents from aqueous media. The bentonite organoclays were found to be more effective than laterite organoclays, however, both can be made easily and can effectively remove the required hydrophobic materials from water and wastewater. Thus they could provide a solution for pollution remediation in Pakistan. The study of organoclays is a large field and shows an immense potential to be explored. It has also been recommended that additional work is required to predict the performance of the adsorption processes for hydrophobic compounds removal from real industrial effluents.

## Figures and Tables

**Table 1:** X-ray fluorescence results of clay mineral.

Elements	Laterite (%)	Bentonite (%)
SiO_2_	28.03	53.80
Al_2_O_3_	23.06	14.98
CaO	9.35	11.39
K_2_O	0.14	4.01
Fe_2_O_3_	36.22	5.84
MgO	3.20	7.45
Na_2_O	0.1	2.40

**Table 2:** Sorption capacities of organoclays used in the study.

Sorbent	Diesel oil (g/g)	Engine oil (g/g)	Edible oil (g/g)
HDTMA-B	5.4	2.89	4.12
HDTMA-L	3.13	1.56	3.33
TDTMA-B	4.7	2.57	3.87
TDTMA-L	2.48	1.11	2.97
